# Increased frequency of follicular helper T cells in mice viral myocarditis is relevant with anti-ANT antoantibody

**DOI:** 10.1186/s12985-015-0257-9

**Published:** 2015-02-11

**Authors:** Fan Yang, Wen-hong Mo, Bao-ping Tan, Xiao-mou Wei, Hong Wang

**Affiliations:** Department of Cardiology, the Fourth Affiliated Hospital of Guangxi Medical University, Liu-Shi Road 1, 545005 Liuzhou, China

**Keywords:** Follicular helper T cells, myocarditis, Anti-adenine nucleotide translocator autoantibody

## Abstract

**Background:**

Recently, a new subset of CD4^+^T helper cell termed Follicular helper T cells (Tfh), which play a pivotal role in B cell activation and differentiation in lymphoid structures, has been reported to participate in some certain autoimmune diseases. But whether Tfh cells are involved in the pathogenesis of VMC remains unclear.

**Methods:**

Male BALB/c mice were intraperitoneally (i.p) infected with CVB3 to establish VMC models. Control mice were treated with phosphate-buffered saline i.p. On 0, 1, 2, 3, 4, 6 weeks post injection, frequencies of splenic Tfh cells were determined by flow cytometric analysis, productions of IL-21 and anti-adenine nucleotide translocator(ANT) autoantibody were detected by enzyme-linked immunosorbent assay. To further investigate the effects of Tfh cells, VMC mice were treated with Anti-IL-21 neutralizing antibody. Heart pathology was examined histologically, the frequencies of Tfh cells and the expressions of anti-ANT autoantibody were investigated after anti-IL-21 intervention. Spearman analysis was used to evaluate the relationship between the frequencies of Tfh cells and IL-21 levels with anti-ANT autoantibody.

**Results:**

The percentage of Tfh cells significantly increased in VMC mice from 1 W to 6 W, the serum level of IL-21 and ANT autoantibody were also significantly increased in VMC mice. Neutralization of IL-21 with anti-IL-21 can ameliorate the myocardium inflammation, decrease Tfh cells and ANT autoantibody after IL-21 antibody intervention compared with those of the control (*P* < 0.05). Both of the frequencies of Tfh cells and IL-21 levels were positively correlated with anti-ANT antibody levels (R = 0.758, *P* < 0.05; R = 0.88, *P* < 0.01, respectively).

**Conclusions:**

Those results suggest that Tfh cells and IL-21 might involve in the pathogenesis of VMC and play an important role in anti-ANT autoantibody production. Targeting the Tfh cell and IL-21 may be a new therapeutic target for the treatment of CVB3-induced VMC.

## Background

Viral myocarditis(VMC) is an autoimmune disease and often progresses to chronic myocarditis, dilated cardiomyopathy (DCM) [1]. Excessive immune responses (mediated especially by activated CD4^+^ T lymphocytes) are the dominant causes of myocardial cell damage. Humoural immune responses following VMC infection are also significant in the pathogenesis of VMC infection. Current knowledge on the role of T responses in the pathogenesis of myocarditis including T helper (Th)1 [2,3], Th17 [4-7] and Th22 response [8]. What more, assisting autoantibody, produced by activated CD4^+^T cells is a fundamental respect of immune responses. Anti-β1-adrenergic receptor autoantibodies, anti-adenine nucleotide translocator(ANT) autoantibodies and anti-cardiac myosin autoantibodies were common seen in VMC and DCM patients, suggesting the existence of abnormal humoral autoimmune responses in VMC. Among these autoantibodies, anti-ANT autoantibodies, whose antigenic determinant is similar with Coxsackievirus B3 play a significant role in the cardiac damages of viral myocarditis [9].

Follicular T helper cells (Tfh), as a separate subset of CD4^+^T helper cells and one of the most significant subsets of effector T cells in lymphoid tissues, have recently emerged [10]. The major function of Tfh cells is to aid B cells during GC reactions and antibody production during humoral immune response [11]. Distinguishing features of Tfh cells include CXC chemokine receptor 5 (CXCR5) expression, inducible co-stimulator (ICOS), programmed death 1(PD-1), and the secretion of interleukin (IL)-21(IL-21). IL-21, which stimulates B cells to proliferation and differentiate into antibody-forming cells via the IL-21 receptor, was a cytokine preferentially excreted by Tfh cells [12,13]. Abnormal Tfh cells frequency and regulation of Tfh cell function could contributes to the pathogenesis of autoimmune-related diseases [14]. Anti-ANT autoantibodies are related to the development of Coxsackievirus B3 (CVB3)-triggered VMC. However, little is known about the potential role of Tfh cells and the relationship between Tfh cells with Anti-ANT autoantibodies in VMC. Therefore, the present study aimed to clarify whether Tfh cells involving in the pathogenesis of VMC and to determine whether Tfh cells play an important role in pathogenic anti-ANT autoantibody production in VMC. Besides, the IL-21 monoclonal antibody (mAb) was given to VMC mice after CVB3 infection to investigate whether IL-21 is associated with the the production of anti-ANT autoantibodies in VMC mouse model.

## Results

### Increased Tfh cells and IL-21 in VMC mice

**Figure 1:**
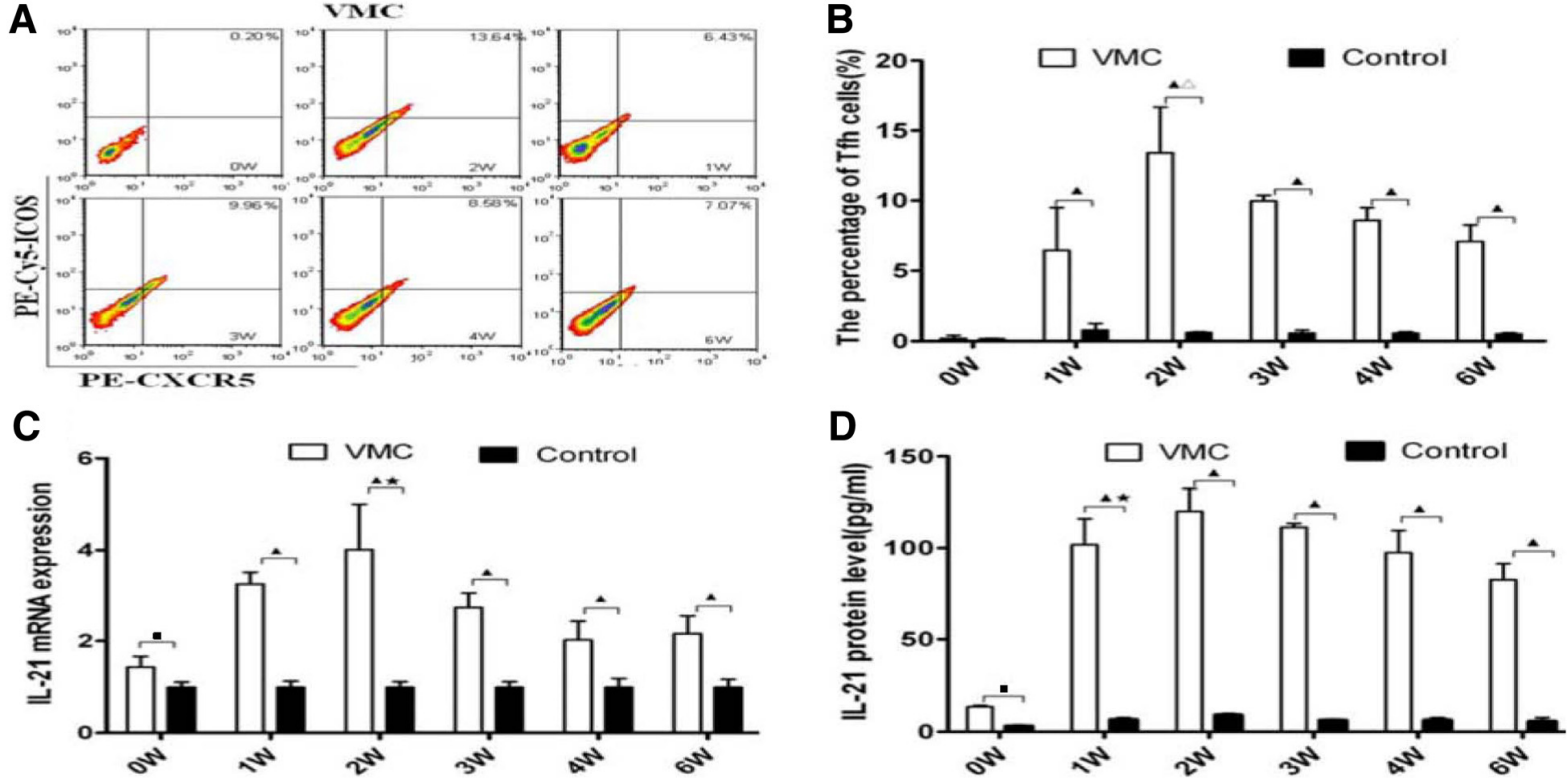
**Elevated Tfh cells and IL-21 in VMC. A**. Representative pictures of CXCR5^+^ICOS^+^CD4^+^ Tfh cells in VMC mice. Numbers in the upper right quadrants indicate the mean percentages of Tfh cells in VMC mice. All staining samples were restimulated with PMA/ionomycin for 4 h before analysis. **B**. The results of the Tfh cells statistical analysis. ^▲^ *P* < 0.05, versus control group. ^Δ^ *P* < 0.01, versus week 0, 1, 3, 4, and 6 VMC mice. **C**. Transcription levels of IL-21 in myocardium. IL-21 mRNA transcripts were higher, in the VMC group than in the PBS group. ^▲^ *P* < 0.05, versus control group. ^■^ *P* <0.05, versus week 1, 2, 3, 4, and 6 VMC mice. ^★^ *P* <0.05, versus week 0, 3, 4, and 6 VMC mice. **D**. IL-21 protein levels in serum. IL-21 protein levels were steadily increased in the VMC mice from 1 week after i.p, and reached statistical difference comparing with those of controls. The highest level of IL-21 in VMC occurred on 2nd week. ^▲^ *P* < 0.05, versus control group. ^■^ *P* <0.05, versus week 1, 2, 3, 4, and 6 VMC mice, ^★^ *P* <0.05, versus week 0, 2, 3 and 6 VMC mice.

The percentages of Tfh cells in the spleens of VMC mice and controls were analyzed by flow cytometry (FCM). The percentages of CXCR5^+^ICOS^+^cells among the CD4^+^T lymphocytes were used to represent the Tfh cells (CXCR5^+^ICOS^+^CD4^+^). There was a significant increase in the percentage of Tfh cells in the VMC mice as compared with those of controls from the first week after viral infection. Tfh cells frequencies in each subgroup of VMC and control mice were 0.20 ± 0.11% vs 0.16 ± 0.02%(0 W), 6.43 ± 3.05% vs 0.79 ± 0.50%(1 W), 13.44 ± 3.25% vs 0.61 ± 0.07%(2 W), 9.96 ± 0.42% vs 0.57 ± 0.23%(3 W), 8.58 ± 0.90% vs 0.56 ± 0.23%(4 W) and 7.07 ± 1.17% vs 0.52 ± 0.17%(6 W) [Figure 1A, B]. It becomes clear that IL-21 produced by Tfh cells serve as an important regulator of humoral responses. Thus we detected the IL-21 mRNA in the myocardium by RT-PCR and protein levels in the serum by ELISA. IL-21 transcripts were abundant in all samples from the first to the sixth week in VMC mice [Figure 1C]. The highest level was reached on the second week. IL-21 mRNA transcripts were 1.04 ± 0.22, 3.25 ± 0.26, 4.01 ± 0.98, 2.73 ± 0.32, 2.02 ± 0.41 and 2.16 ± 0.39 times higher respectively in the VMC group than in the PBS group. There was a significant increase of IL-21 protein levels in VMC mice as compared with those in the PBS controls from the 1^st^ week to 6^th^ week. The highest level was reached on 2^nd^ week. The IL-21 protein levels at different times in VMC and control mice were 13.73 ± 0.75 vs 13.07 ± 0.96 pg/ml, 101.81 ± 14.01 vs 13.02 ± 1.14 pg/ml, 119.84 ± 12.46 vs 22.21 ± 6.26 pg/ml, 111.37 ± 1.90 vs 34.08 ± 4.44 pg/ml, 97.54 ± 12.14 vs 30.29 ± 18.64 pg/ml and 82.61 ± 8.78 vs 36.20 ± 6.23 pg/ml respectively [Figure 1D].

### Increased levels of anti-ANT autoantibody in VMC mice

**Figure 2:**
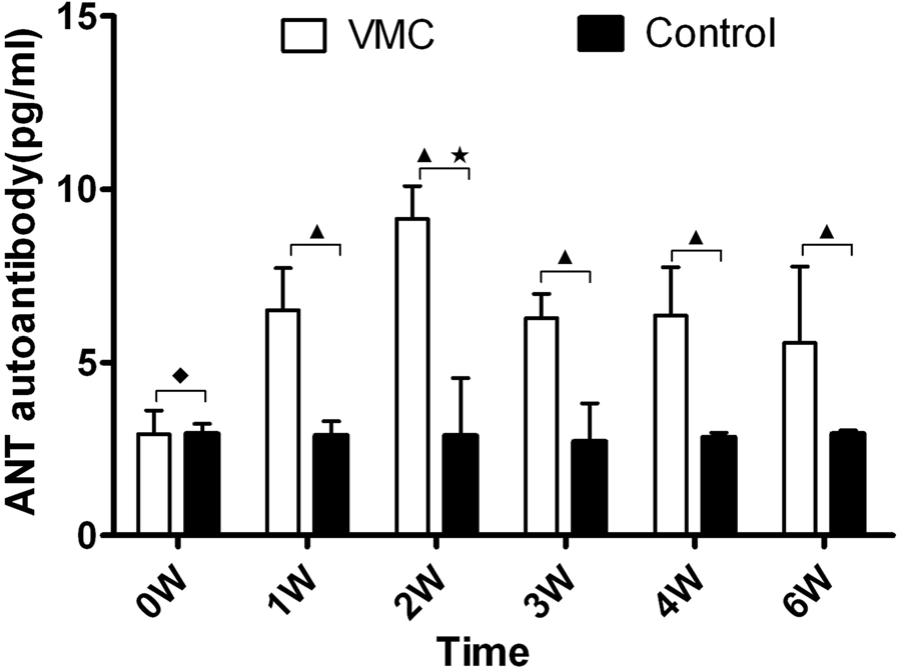
**Anti-ANT autoantibody levels increased in the serum of VMC mice.** ^▲^ *P* < 0.05, versus control group. ^◆^ *P* <0.05, versus week 1, 2, 3, 4, and 6 VMC mice, ^★^ *P* <0.05, versus week 0, 2, 3 and 6 VMC mice.

From the first week, anti-ANT autoantibody levels in the serum began increasing in a stable manner, peaking on the second week, and possessing high expression till the sixth week [Figure 2]. The anti-ANT autoantibody levels at different times in VMC and control mice were 2.93 ± 0.68 vs 2.94 ± 0.29 pg/ml, 6.51 ± 1.21 vs 2.89 ± 0.41 pg/ml, 9.15 ± 0.95 vs 2.66 ± 1.37 pg/ml, 6.27 ± 0.71 VS 2.71 ± 0.11 pg/ml, 6.35 ± 1.40 vs 2.83 ± 0.14 pg/ml and 5.57 ± 2.20 vs 2.94 ± 0.10 pg/ml separately. Significant statistics difference were seen when comparing anti-ANT autoantibody between VMC and control group except 0 week (*P* > 0.05).

### IL-21mAb alleviated the severity of myocarditis

**Figure 3:**
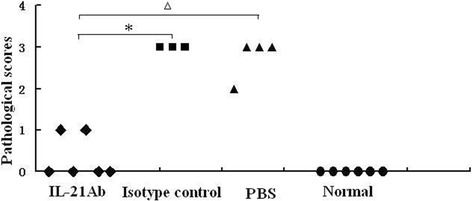
**The pathological scores attenuated after IL-21 inhibition.** Each point represents an individual mouse, *P <0.05, versus isotype control group mice, ^△^ *P* <0.05, versus PBS group mice.

The number of mice who survived to 14d was 6, 6, 3 and 4 for normal, IL-21mAb, isotype control and PBS groups separately. All survival mice were sacrificed on the day 14^th^ after intervention. Histological results showed that IL-21mAb alleviated the severity of myocarditis. The pathological scores of IL-21mAb mice were much lower than isotype control-treated and PBS mice [Figure 3]. The pathological scores of IL-21mAb group mice were slightly higher than those in the normal group, but no statistical difference was seen between them (*P* > 0.05). However, there was no significant difference of the pathological scores of heart section between the isotype antibody-treated groups and PBS groups (*P* > 0.05).

### Blockade of IL-21 reduced Tfh cell proportions and circulating level of anti-ANT autoantibodies

**Figure 4:**
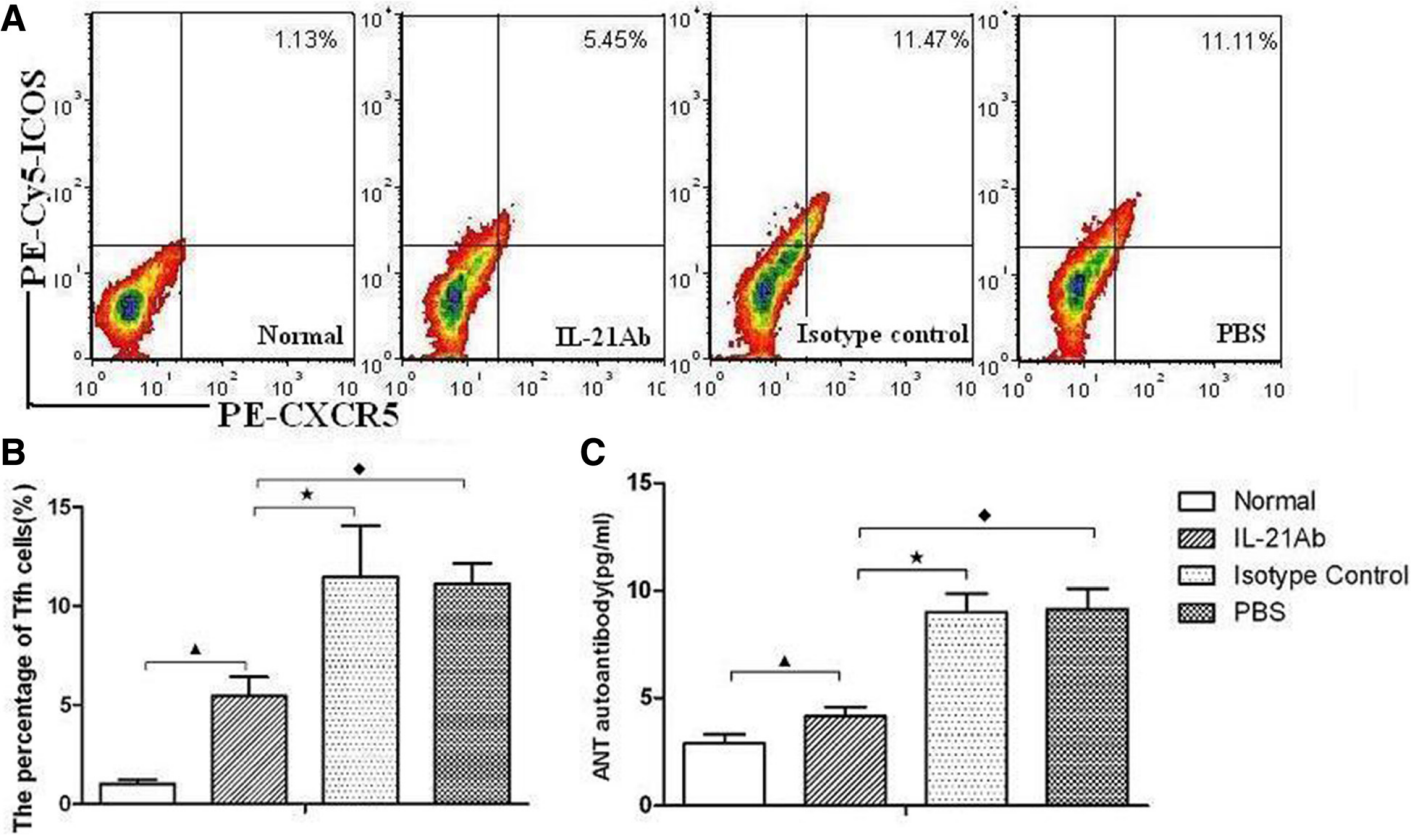
**Tfh cell proportions and circulating level of anti-ANT autoantibodies decreased after IL-21 inhibition. A**. The percentages of Tfh cells in each groups investigated by flow cytometry. Tfh subsets were gated with CXCR5^+^ICOS^+^/CD4^+^ cells. Numbers in upper right quadrants represents an average number of positive cells in each group. All staining samples were restimulated with PMA/ionomycin for 4 h before analysis. **B**. The results of the Tfh cells statistical analysis. The percentages of Tfh cells in IL-21mAb group was fewer than control and PBS group. ^▲^ *P* < 0.05, versus normal group. ^★^ *P* < 0.05, versus isotype control mice. ^◆^ *P* < 0.05, versus PBS group mice. **C**. Anti-ANT autoantibody levels in the different groups. ^▲^ *P* < 0.05, versus normal group. ^★^ *P* < 0.05, versus isotype control mice. ^◆^ *P* <0.05, versus PBS group mice.

Compared with those in the normal group, the percentages of Tfh cells in the IL-21 mAb, isotype control and PBS groups increased markedly (*P* < 0.05) [Figure 4A, B]. The proportion of Tfh cells in the IL-21 mAb group trended lower than that of the isotype control and PBS groups, there was significant difference among them (*P* < 0.05) [Figure 4B]. Tfh frequencies in the IL-21 mAb, isotype control, PBS group and normal were 5.45 ± 0.97%, 11.47 ± 2.57%, 11.11 ± 1.04% and 1.13 ± 0.19% respectively. The levels of serum anti-ANT autoantibody levels in the IL-21 mAb, isotype control and PBS groups were elevated dramatically, compared with those in the normal group, especially in the isotype control and PBS groups [Figure 4C]. The levels of serum anti-ANT autoantibody in the normal group, IL-21 mAb, isotype control and PBS and were 2.89 ± 0.41 pg/ml, 4.15 ± 0.45 pg/ml, 9.15 ± 0.95 pg/ml, 9.01 ± 0.86 pg/ml. Statistical difference were seen when compared the levels of Anti-ANT antibody among these four groups, *P* < 0.05. The levels of serum anti-ANT autoantibody in the IL-21 mAb group were much lower than those in the isotype control and PBS groups (*P* < 0.05) [Figure 4C], which indicated that blockade of IL-21 can reduced circulating level of anti-ANT autoantibodies. No significant difference was seen between the isotype control group and PBS group (*P* > 0.05).

### Positive correlation of Tfh cell proportions and IL-21 with levels of Anti-ANT autoantibody

**Figure 5:**
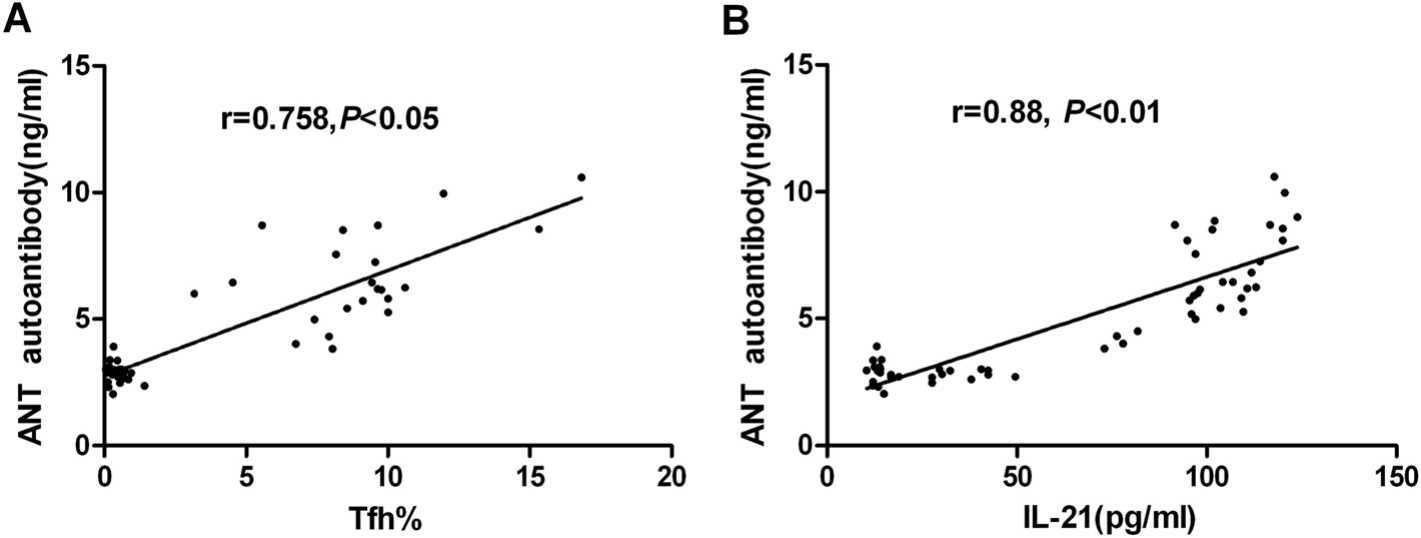
**Aberrant CXCR5** ^**+**^ **ICOS** ^**+**^ **/CD4** ^**+**^ **cells proportions and IL-21 correlated with anti-ANT autoantibody titers in VMC mice. A**. The correlation between percentages of CXCR5^+^ICOS^+^/CD4^+^ cells with anti-ANT autoantibody in VMC mice. **B**. The correlation between levels of IL-21 with anti-ANT autoantibody titers in VMC mice. Spearman correlation was applied to depict linear relationships. Each data point represents an individual subject.

FCM results suggested that Tfh cells and IL-21 increased in VMC mice, and blockade of IL-21 reduced Tfh cell proportions. Thus, we then analyzed the relationship between the anti-ANT autoantibody titers with the percentages of Tfh cells and IL-21 level. The percentages of CXCR5^+^ICOS^+^CD4^+^T lymphocytes showed a positive correlation with the anti-ANT autoantibody titers (r = 0.758, *P* < 0.05) [Figure 5A]. The correlation between the level of IL-21 and the anti-ANT antibody titers was also analyzed, and a similarly positive correlation was observed (r = 0.88, *P* < 0.01) [Figure 5B].

## Discussion

Although VMC has appeared to be a T-cell-mediated autoimmune disease over the past few decades, Neither Th1, Th2 nor Th17 could elucidate the pathogenesis of VMC completely. Besides, high levels of circulating autoantibodies, such as anti-β1-adrenergic receptor autoantibodies, anti- ANT autoantibodies, and anti-cardiac myosin autoantibodies produced by B cells suggested that B cells are also correlated with the development and severity of the VMC [9,15,16]. Previous researches presumed that Th2 cells may play a crucial role in B cell-mediated humoural immune responses. Tfh cells, a new separate CD4^+^ T helper lineage, have attracted close attention for their specialized role in assisting B cells and contributing to autoimmunity [10,17,18]. Tfh cells could provide costimulation signal to promote growth, differentiation, and class switching of B cells and lead to excessive autoantibody production by specific high expression of costimulatory molecules such as ICOS and CD40L. In addition, IL-21, which preferentially produced by Tfh cells and served as an important regulator of humoral responses, could drive B cell expansion and differentiation. Abnormal distribution and frequency of Tfh cells are associated with the pathogenesis of systemic lupus erythematosus (SLE) [19-21], rheumatoid arthritis [22,23], Sjögren syndrome [24,25] and other autoimmune diseases [26,27]. Besides, evidence has shown that excessive production of IL-21 led to autoimmunity [28,29]. However, their association with VMC remains largely unknown.

Anti-ANT autoantibody is a cross-reacting antibody against ANT at the inner mitochondrial membrane of myocardium, whose antigenic determinants are similar with those of CVB3 [27]. High titer of this autoantibody has been recognized as a marker of the severity and the activity of the VMC [27]. The present study aimed to determine whether Tfh cells play a pathogenic role in VMC and to clarify their involvement in the anti-ANT autoantibodies production of VMC. The increased frequency of Tfh cells and level of IL-21 were detected in VMC mice induced by CVB3. These results indicated the possible involvement of Tfh cells and IL-21 in the pathogenesis of VMC. More importantly, inhibition of aberrant circulating IL-21 by neutralizing Ab can decreased the proportion of Tfh cells and levels of anti-ANT autoantibodies. These are in agreement with the studies of Tfh cells are related to autoantibody production in other autoimmune diseases [30,31]. Consistent with these data, we describe here that Tfh cells and IL-21 concentration have a significantly positive correlation with anti-ANT autoantibody level. These data also indicated that IL-21 is a promoting factor in the differentiation/expansion of Tfh cells, and antibody production, in murine model of VMC. Our research showed that blocking IL-21 signaling pathway in VMC mice led to attenuate inflammation of myocardium and production of pathogenic anti-ANT autoantibody, which indicates that IL-21 pathway might be a potential treatment for VMC.

However, it is worth noting that IL-21 are also expressed by Th17 cells [32,33]. Thus, the finding of increased IL-21 is not exact proof of Tfh activation but could also be a result of Th17 activity. Yet it were Tfh cells but not Th17 cells might be the main producer of IL-21 that contribute to the VMC processes, as the time course of Th17 cells do not coincidence with that of IL-21 [4]. In addition, Studies have disclosed a subset with a overlapping features of Tfh -cells [11,34], named Th17-like Tfh cell. To deplete the contribution of Th17-like Tfh subsets in VMC evolvement, spleen Th17-like Tfh cell subsets were defined by multicolor flow cytometry (CD4^+^ICOS^+^CCR6^+^). No significant difference of Th17-like Tfh frequency was observed in VMC mice compared to control individuals (data unpublished).

## Conclusion

Taken together, our results indicated that Tfh cells were involved in the pathogenesis of VMC and that they may play a role in the production of pathogenic anti-ANT autoantibodies. Meanwhile, blockade of IL-21 can inhibit Tfh cell and anti-ANT autoantibody secretion in vivo. As exact causality between Tfh cells and anti-ANT autoantibody have not clarified. Further studies are needed to determine how Tfh cells initiate anti-ANT autoantibody production in VMC and to clarify the mechanisms involved.

## Materials and methods

### Mice and ethics statement

Four-week-old SPF male BALB/c mice were obtained from Shanghai Laboratory Animal Center, Chinese Academy of Sciences. All animals were housed under pathogen-free conditions at the Experimental Animal Center of the Guangxi Medical University. Experiments using mice were performed in accordance with protocols approved by Guangxi Medical University Animal Ethics Committee.

### CVB3 titration and myocarditis induced

Heart-passaged CVB3 (Nancy strain) was propagated in Hep-2 cells, the 50% tissue culture infectious dose (TCID_50_) titer was determined by the cytopathic effects visible after 72 h. The TCID_50_ assay result for Hep-2 cells was 1 × 10^−7^. BALB/c mice were infected by intraperitoneal (i.p.) injection of 0.1 ml of PBS containing approximately 100TCID_50_ of the virus (VMC group n = 60, 10 mice/subgroup) or inoculated i.p. with PBS(control group, n = 60, 10 mice/subgroup). Each group was divided into 6 subgroups (Week 0, 1, 2, 3, 4 and 6). The day of injection was defined as week 0. Surviving mice of 6 subgroups were separately sacrificed by cervical dislocation on 0, 1, 2, 3, 4, and 6 weeks after i.p. injection.

### Histopathology

The ventricular tissues of the hearts were cut longitudinally, fixed in 10% formalin, then embedded in paraffin, and stained with hematoxylin & eosin, histopathological change was observed by using light microscopy (Nikon Eclipse E800 Microscope, Kawasaki, Kanagawa, Japan). Pathological scores were graded by two independent pathologists separately in a blinded manner based on the following semi-quantitative scale: 0, no inflammatory infiltrates; 1, small foci of inflammatory cells between myocytes or inflammatory cells surrounding individual myocytes; 2, larger foci of 100 inflammatory cells or involving at least 30 myocytes; 3, 10% of a myocardial cross-section involved; 4, 30% of a myocardial cross-section involved [35].

### Lymphocyte preparation

Spleens from virus-infected mice and controls were harvested aseptically. The lymphocyte fractions of these samples were obtained by Ficoll Plaque (Solarbio Science & Technology, China) gradient centrifugation. Lymphocytes were maintained in a 24-well flat-bottom tissue culture plate with RPMI 1640 supplemented with 10% fetal calf serum (Gibco, USA) at 37°C in a humidified atmosphere with 5% CO_2_.

### Flow cytometry

Lymphocytes at 10^6^/tube were stained with fluorescein isothiocyanate (FITC)-conjugated anti-CD4, phycoerythrin (PE)-conjugated anti-CXCR5, phycoerythrin-Cy5 (PE-Cy5)-conjugated anti-ICOS (BD PharMingen San Diego, CA) or isotype-matched control IgG, according to the manufacturer’s protocol and fixed in 4% paraformaldehyde. Flow data were analyzed on a FACS Calibur flow cytometer (BD Biosciences). CellQuest software (BD Biosciences) was used for data acquisition. At least 50,000 events per sample were analyzed. Cells were gated on the forward scatter of living cells and then centered on CD4^+^T cells. After setting the threshold using isotype control staining for CXCR5 and ICOS, the proportion of Tfh cells was analyzed as the percentage of CXCR5^+^ICOS^+^cells within total CD4^+^T cells for each sample, and percentages of CXCR5^+^ICOS^+^/CD4^+^cells proportion was defined as Tfh cells.

### Real-time RT–PCR

Total RNA including the small RNA fraction of homogenized heart tissues was extracted with TRIZOL Reagent® (Invitrogen, USA), and then reverse transcripted into cDNA with an Reverse Transcription kit (Ferma, CA) according to the manufacturer’s instructions. Primer for IL-21 and the housekeeping gene GAPDH are designed by Primer Premier 5.0. Real time-polymerase chain reaction (RT-PCR) was performed using an ABI 7500 Sequence Detection System (Applied Biosystems, Foster City, CA) using SYBR green. The amplification steps consisted of denaturation at 95°C, followed by 40 cycles of denaturation at 95°C for 15 s and then annealing at 60°C for 1 min. The relative gene expressions were normalized to the level of GAPDH transcripts and quantified by the ^∆∆^CT method using 7500 System Sequence Detection software (Applied Biosystems). All reactions were performed in at least triplicate for each sample.

### Cytokine assay

Blood was collected via retro-orbital bleeding, and serum was separated. The amounts of IL-21 and ANT autoantibody in the blood serum were detected using the Quantikine Mouse IL-21 (R&D Systems, Minneapolis, MN) and ANT Immunoassay (CUSABIO, China). No cross-reactivity was observed in detection. All samples were measured in triplicate.

### Interventions and groups

Mice infected with CVB3 (VMC mice) were separated into three groups: administered either IL-21mAb (100 μg per mouse, eBioscience, IL-21 mAb group, n = 6), isotype control immuno-globulin (Ig) G1Ab (100 μg per mouse, eBioscience, isotype control group, n = 6), or PBS (PBS group, n = 6) i.p. at day 0,4,7 and 10 after CVB3 infection. In addition, BALB/c mice without any intervention were assigned as the normal control (normal group, n = 6). All animals were killed on day 14 after virus infection. The values of the pathological scores were recorded. The percentages of CXCR5^+^ICOS^+^/CD4^+^cells proportion and the amounts of anti-ANT autoantibody in the blood serum were recorded at the same time.

### Statistical analysis

Data were shown as the mean ± SD. Statistical analysis of the data was performed with one-way ANOVA using SPSS17.0. Bivariate correlation was used as a test of correlation between two variables, *P* < 0.05 was considered statistically significant.
